# Characterization of the complete chloroplast genome sequence of the endangered species *Platycrater arguta* (Hydrangeaceae)

**DOI:** 10.1080/23802359.2020.1821816

**Published:** 2020-09-21

**Authors:** Yue-Ling Li, Chun-Yan Wei, Xing-Yu Chen, Zhong-Shuai Sun

**Affiliations:** a Zhejiang Provincial Key Laboratory of Plant Evolutionary Ecology and Conservation, Taizhou University, Taizhou, P. R. China; b College of Life Sciences, Taizhou University, Taizhou, P. R. China

**Keywords:** *Platycrater arguta*, endangered species, chloroplast genome, Phylogenomics

## Abstract

*Platycrater arguta* is a rare and endangered shrub species endemic to East Asia. Here, we report the complete chloroplast (cp) genome structure and its taxonomic position within Hydrangeaceae to promote its conservation and restoration. The complete cp genome of *P. arguta* was 157,810 bp in length and contained a large single-copy region (LSC) of 86,823 bp and a small single-copy region (SSC) of 18,735 bp, as well as a pair of inverted repeat (IR) regions of 26,126 bp, each. 113 unique genes are predicted in this cp genome, including 79 protein-coding genes, 30 transfer RNA (tRNA) genes and 4 rRNAs. Maximum-likelihood (ML) phylogenetic analysis based on 79 shared cp CDS (coding DNA sequences) of 19 species reveals a close relationship between *P. arguta* and *Schizophragma hydrangeoides.*

*Platycrater* Sieb. et Zucc. is a monotypic genus in Hydrangeaceae that includes *Platycrater arguta*, a rare and endangered species endemic to East Asia (Fu 1989; Ao 2008). Studies on this rare and disjunct distributed temperate shrub are significant in understanding of the evolutionary history of Sino-Japanese Floristic Region (Qiu et al. 2009; Qi et al. 2014). Because of its small range size and small number of populations, the species has been considered ‘threatened’ by the China Species Red List (Wang and Xie 2004), and it was involved in the priority list of species of the greatest conservation concern in the Chinese Biodiversity Action Plan in 1994 (Ao 2008; Qiu et al. 2009). The classification of the Hydrangeaceae has long been problematic, and phylogenetic studies using a limited set of markers have often not been able to fully resolve relationships among this genus (Hufford et al. 2001). By taking advantages of next-generation sequencing technologies that efficiently provide the chloroplast (cp) genomic resources of our interested species, we can rapidly access the abundant genetic information for phylogenetic research and conservation genetics (Liu et al. 2017; Chen et al. 2019). Therefore, we sequenced the whole cp genome of *P. arguta* to elucidate its phylogenetic relationship within Hydrangeaceae.

Total genomic DNA was extracted from silica-dried leaves collected from Niutoushan, Linhai City (Zhejiang, China) using a modified CTAB method (Doyle and Doyle 1987). A voucher specimen (Li18001) was collected and deposited in the Herbarium of Taizhou University. Sequencing was conducted on the HiSeq 2500 platform. We assembled the cp genomes with NOVOPlasty (Dierckxsens et al. 2017) and annotated with the dual organellar genome annotator (DOGMA; Wyman et al. 2004). Then, BLAST was used to check the annotation, followed by manual correction through comparison with other closely related cp genomes of Hydrangeaceae in Geneious R11 (Biomatters Ltd., Auckland, New Zealand).

The complete cp genome of *P. arguta* (GenBank accession MT610904) was 157,810 bp long consisting of a pair of inverted repeat (IR) regions (26,126 bp, each) divided by large single-copy (LSC) and small single-copy regions (LSC) of 86,823 bp and 18,735 bp, respectively. The overall GC contents of the total length, LSC, SSC, and IR regions were 37.8, 36.0, 31.5, and 43.1%, respectively. A total of 113 unique genes were predicted and annotated, including 79 protein-coding genes, 30 transfer RNA (tRNA) genes, and four rRNAs. Among the 113 genes, three genes crossed adjacent regions: the *ycf1* gene crossed the SSC/IRa junction, the *rps19* gene crossed IRb/LSC junction, and ndhF crossed the IRb/SSC junction.

To investigate the taxonomic positions of *P. arguta*, a maximum-likelihood (ML) analysis was performed using RAxML-HPC2 version 7.6.3 (Stamatakis 2006) at the CIPRES Science Gateway (http://www.phylo.org/) using a cp CDS (coding DNA sequences) matrix consisting of CDS sequences of 19 Hydrangeaceae species and two outgroup taxa. The ML tree (Figure 1) was consistent with the most recent phylogenetic study on Hydrangeaceae (Soltis et al. 1995; Hufford et al. 2001). According to the phylogenetic tree, *P. arguta* belongs to tribe Hydrangeeae of subfamily Hydrangeoideae and closely related to *Schizophragma hydrangeoides* Sieb. et Zucc.

**Figure 1.**
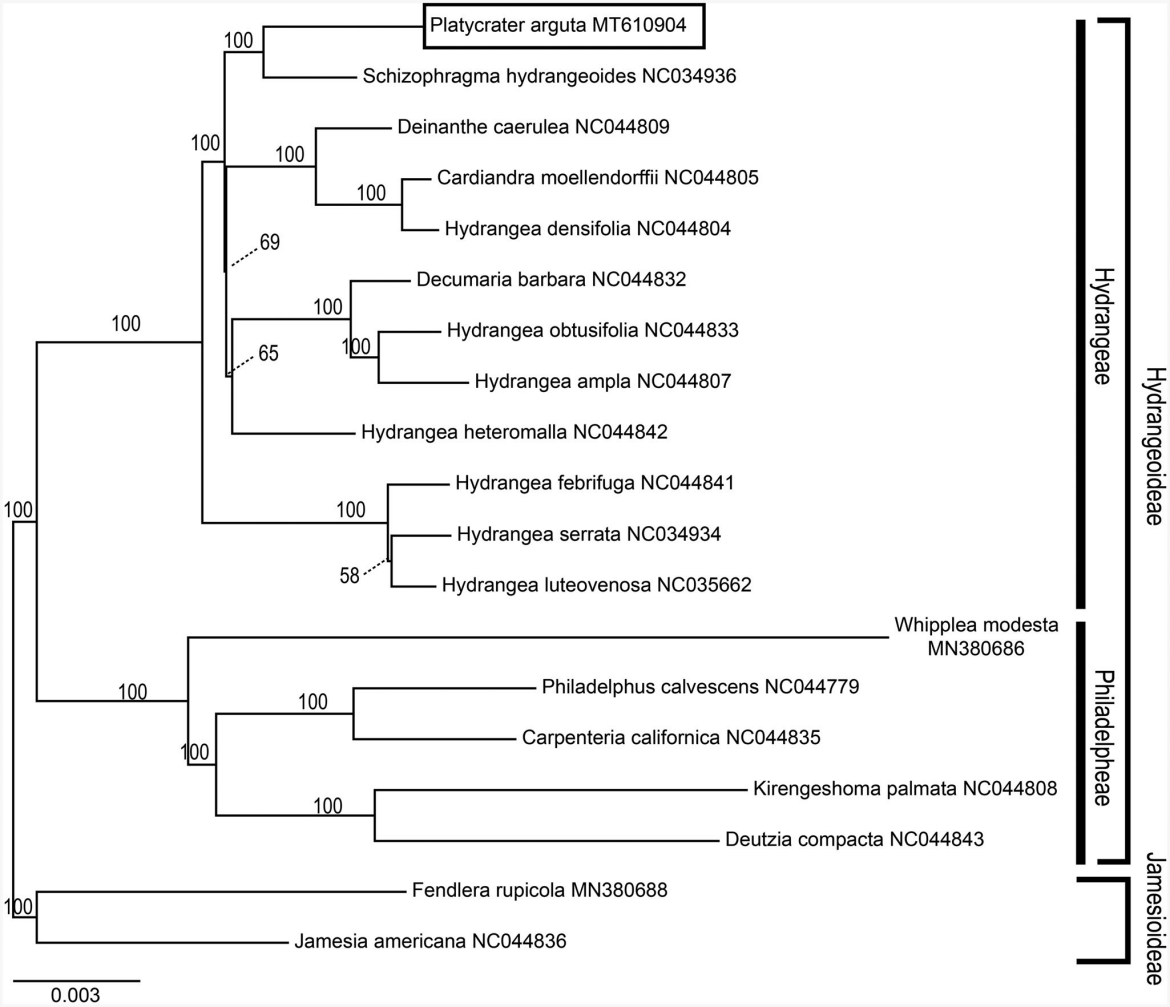
ML phylogenetic tree based on concatenated chloroplast CDS sequences of 19 species from Hydrangeaceae. Relative branch lengths are indicated. Numbers above each branch are the support values. Accession numbers are written behind species names.

## Data Availability

The data that support the findings of this study are openly available in GenBank of NCBI at https://www.ncbi.nlm.nih.gov, reference number MT610904. The raw sequencing reads used in this study were deposited in the Sequence Read Archive (SRA) under accession number PRJNA659226.
